# Two-Photon Imaging Reveals a Power Threshold of Neuronal Activation by Electrochemical Sensing Waveforms

**DOI:** 10.1021/acs.analchem.6c03024

**Published:** 2026-08-31

**Authors:** Nathaniel P. Williams, Elaine M. Robbins, Anna M. Kelly, Alberto L. Vazquez, X. Tracy Cui

**Affiliations:** † Department of Bioengineering, 6614 University of Pittsburgh, Pittsburgh, Pennsylvania 15261, United States; ‡ Center for Neural Basis of Cognition, Pittsburgh, Pennsylvania 15213, United States; § Department of Radiology, University of Pittsburgh, Pittsburgh, Pennsylvania 15261, United States; ∥ University of Pittsburgh McGowan Institute for Regenerative Medicine, Pittsburgh, Pennsylvania 15261, United States

## Abstract

Electrochemical sensing enables high-temporal-resolution measurements of neurochemical dynamics, yet the impact of sensing waveforms on local neural excitability has not been systematically examined. We combined in vivo two-photon Ca^2+^ imaging in Thy1-GCaMP6s mice with sensing waveform application through carbon fiber microelectrodes implanted in cortex to determine how sensing waveforms affect neuronal activation. Standard fast-scan cyclic voltammetry (FSCV) waveforms for dopamine and serotonin, as well as square-wave voltammetry and amperometry waveforms, produced Ca^2+^ activity indistinguishable from no-stimulation controls even over 30 min, confirming established parameters are physiologically silent. We then systematically explored parameters often proposed to increase sensitivity, enhance specificity, or detect new analytes. Increasing the anodic switching potential of the dopamine FSCV waveform produced strong neuronal activation beginning at ∼2.1 V with rapid saturation in activation area beyond ∼2.3 V. Anodic excursions produced stronger activation than cathodic excursions, and slower scan rates and higher scan frequencies increased activation. Unlike conventional microstimulation, the response to sensing waveforms was delayed by 52 ± 26 s and persisted after waveform cessation, consistent with the diffusion of electrochemical reaction byproducts rather than direct membrane depolarization. These results define a parameter space, corresponding to a power dose of <0.5 mW, where FSCV does not perturb neural activity. This establishes a practical threshold to guide waveform development and help avoid unintended neural activation during electrochemical sensing.

## Graphical abstract

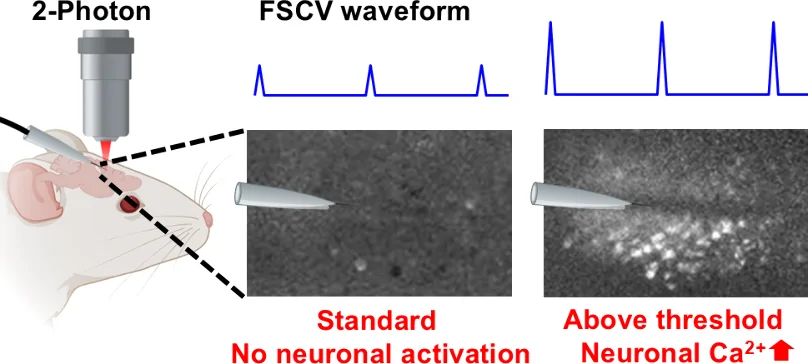

## Introduction

Measuring the dynamics of neurochemical signaling in the living brain is fundamental to understanding how neural circuits regulate behavior, cognition, and disease. Real-time neurotransmitter measurements have illuminated mechanisms underlying reward learning,^1,2^ motor control,^3^ and psychiatric conditions such as depression^4,5^ and addiction^6^ with dynamics that unfold on subsecond and submillimeter scales. Clinically, the ability to monitor neurochemicals directly in the brain holds promise for guiding intraoperative decision-making during neurosurgery^7,8^ and tracking neurochemical biomarkers in traumatic brain injury.^9,10^ Meeting the demands of in vivo neurochemical monitoring requires techniques capable of detecting rapid, spatially localized changes in neurotransmitter concentration in vivo. Electrochemical sensors are uniquely positioned to fulfill these requirements, owing to their direct, reagent-free detection of electroactive species with high temporal and spatial resolution at implanted microelectrodes. Among electrochemical approaches, fast-scan cyclic voltammetry (FSCV), square-wave voltammetry (SWV), and amperometry have become the most widely adopted strategies for in vivo neurochemical measurements. FSCV has been used extensively for decades to investigate many neurotransmitters^11–16^ and has greatly contributed to our understanding of neurochemical signaling. It has been used in awake^1^ and anesthetized animal models of disease^3,17^ as well as in clinical applications.^18^ FSCV sensing of neurotransmitters involves sweeping an optimized voltage waveform quickly (generally 100–1000 V/s) in microelectrodes, generally carbon fiber microelectrodes (CFMEs).^19,20^ The rapid scan rates employed by FSCV offer several advantages: First, each scan is completed very quickly, typically in under 10 ms, so the scans can be applied in rapid succession enabling measurements with high temporal resolution. Second, analyte-derived faradaic current increases with the square root of the scan rate, as shown in eq 1 (see Materials and Methods), where *n* is the number of electrons involved in the reaction, *A* is the electrode area, *D*_O_ is the diffusion coefficient of the analyte undergoing oxidation, *C*_O_* is the bulk analyte concentration, and *v* is the scan rate. The measured current increases with increasing scan rate, leading to increased sensitivity. Finally, neurochemicals with overlapping oxidation potentials can be resolved based on the kinetics of their oxidation reactions. With this technique, electroactive compounds can be detected with reasonable specificity at high temporal (subsecond) and spatial (μm-scale) resolutions in vivo. By optimizing waveform parameters, including scan rate, holding potential, voltage maximums and minimums, and application frequency, FSCV has been used to detect many neurochemicals, including dopamine,^21–25^ serotonin,^26–28^ histamine,^29^ adenosine,^30–32^ and glutamate.^33,34^ Recently, CFME arrays and glassy carbon microelectrode arrays have been developed to detect neurotransmitters at multiple locations simultaneously.^35–39^

The rapid scan rate of FSCV, in addition to increasing analyte-derived faradaic current, also increases non-faradaic current. While faradaic current increases with the square root of the scan rate, non-faradaic current increases directly with scan rate, as shown in eq 2 (see Materials and Methods) where *C*_d_ is the capacitance of the electric double layer. This means that, depending on electrode size, scan rate, and other factors, the generated non-faradaic current may be upward of 1000-fold higher than the current derived from analyte oxidation (See Figure 1D). This necessitates the subtraction of background non-faradaic current, as well as faradaic current from any unchanging electroactive species, to obtain information about the desired neurochemical. In some FSCV software, background subtraction is done automatically before data is displayed to the user, somewhat obscuring the actual amplitude of the current being generated. Despite the widespread and diverse in vivo applications of FSCV, the effects of varying waveform parameters on the excitability of sensitive neural tissue have not been systematically investigated. It remains unclear whether the FSCV waveform itself may alter the activity of nearby neurons, especially as experimenters continue to explore more intensive waveform applications. If the waveform were to induce neurotransmitter release, the measured signal could be confounded. Neurochemical sensing via FSCV is still a rapidly evolving field,^40,13^ where scan rates,^41,42^ voltage ranges,^43–46^ waveform shapes,^47^ and application frequencies^48^ have all been altered to optimize FSCV techniques for various analytes of interest. Some prior research has explored these issues empirically, notably Kirkpatrick et al.^49^ who found only modest changes to membrane potential with patch clamp during FSCV application, on the order of ∼2.5 mV, far less than the ∼20–30 mV change necessary for action potential induction. This effect was observed for a relatively conservative dopamine detection waveform of −0.4 V holding potential with an upper limit of 1.0 V, scanned at 600 V s^–1^, and repeated at 5 Hz. As new sensing waveforms are being explored, it is important to thoroughly investigate the effects of all optimizable FSCV parameters on the neural tissue itself. To do so, we began by applying several published waveforms used to detect neurochemicals in vivo and measuring the neuronal Ca^2+^ response via 2-photon (2P) microscopy in Thy1-GCaMP6s transgenic mice to visualize neural activity before, during, and after FSCV waveform application. We followed this initial investigation by systematically altering scan rate, waveform application frequency, voltage maximum (switching potential), and the duration of the sensing waveform application to isolate the effects of individual parameters on the neural response. We also examined other in vivo controlled-voltage sensing techniques, such as amperometry and SWV. With these experiments, we endeavored to create guidelines for the design of new neurochemical sensing waveforms avoiding undesirable activation of neural tissue that could confound sensing experiments.

**Figure 1:**
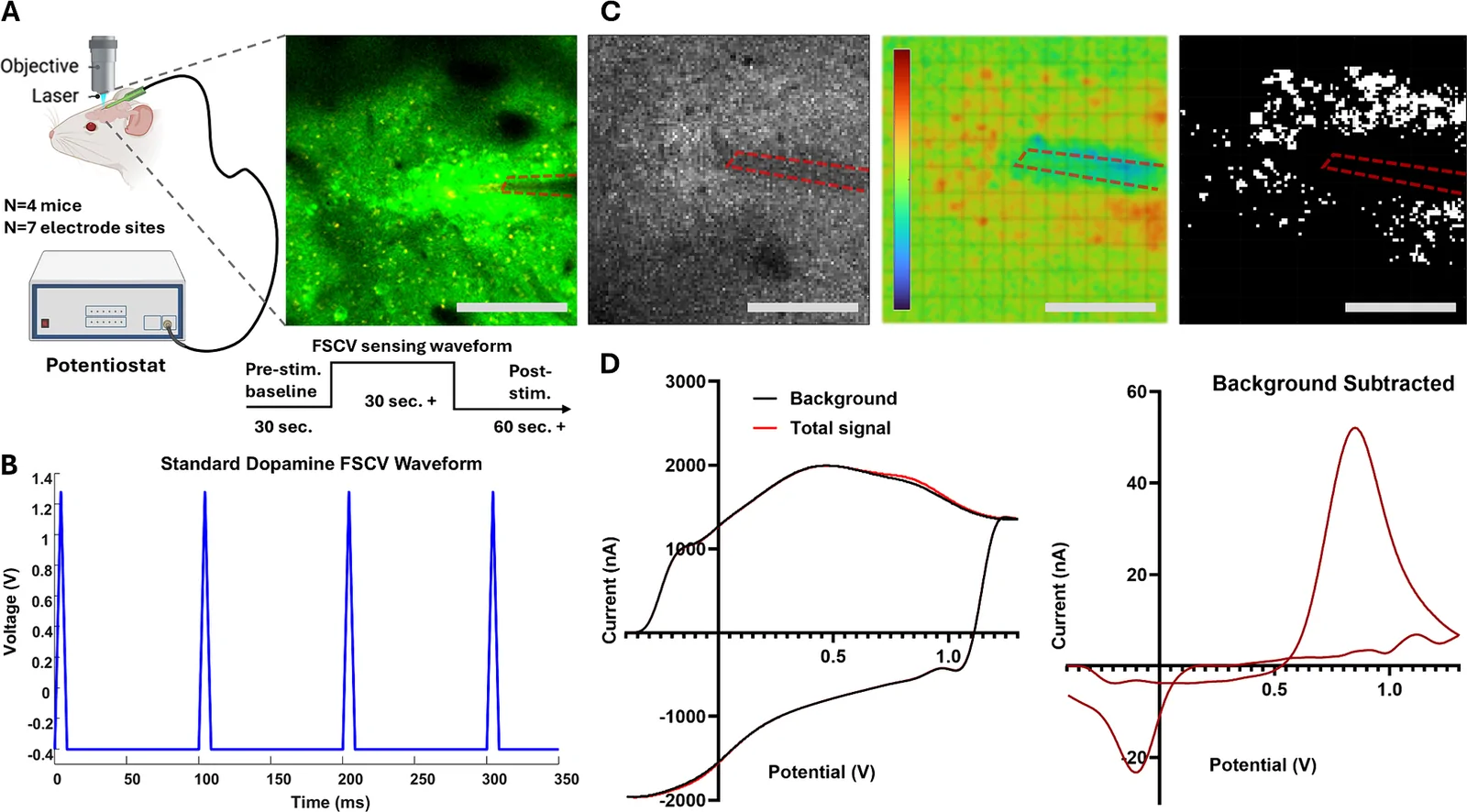
Simultaneous in vivo 2-photon imaging and application of neurochemical detection waveforms. A) Left: Schematic of the experimental setup showing the mouse under the 2-photon objective and the potentiostat connected to the implanted CFME. Right: Example average projection of a suprathreshold neurochemical sensing waveform application. The red dashed outline shows the location of the electrode, and the time course of stimulation is shown below the average projection image. Scale bar = 100 μm. For all data presented in these studies, *n* = 7 electrode penetrations were used across *n* = 4 mice. B) Example voltage trace of the standard dopamine detection waveform showing each element: reversal potential (1.3 V), scan rate (400 V/s), hold (−0.4 V), scan duration (8.5 ms, a function of the scan rate and switching potential), and hold duration (91.5 ms, a function of the scan frequency; 10 Hz, and the scan rate). C) Example images of a stimulation trial. Left: Average fluorescence image for a single stimulation. Center: heatmap of the *z*-score for each pixel. Right: Binary map of pixels (white) that passed the *z*-score threshold. Scale bar = 100 μm. D) Example cyclic voltammogram of the standard dopamine waveform, showing the large nonfaradaic current (left, black) that is subtracted from the total signal (left, red) resulting in the observed dopamine deteciton peak (right).

## Materials and Methods

### CFME Construction

CFMEs were fabricated as previously described.^50–53^ A 7 μm diameter single carbon fiber (Goodfellow USA, Pittsburgh, PA, USA) was threaded through a borosilicate capillary (Sutter Instruments, Novato, CA, USA) using acetone aspiration. The capillaries were pulled to a sharp tip with a capillary puller (Narishige, Los Angeles, CA, USA). The carbon fibers were sealed in place using Spurr low-viscosity epoxy (Sigma-Aldrich) and cured in an oven at 70 °C for 8 h. The exposed carbon fiber was then cut to a length of 400 μm with a scalpel. Mercury (electronic grade, Sigma-Aldrich) was backfilled into the capillary, and a nichrome wire was inserted into the mercury to allow an electrical connection between the potentiostat and the carbon fiber. *Caution: mercury is a toxin that poses a hazard through inhalation as well as skin contact. Mercury handling was performed in a fume hood, using nitrile gloves and secondary containment. Mercury-containing electrodes and waste were stored in sealed, labeled containers and disposed of as hazardous waste in accordance with institutional Environmental Health and Safety guidelines. Any mercury spill was managed using a mercury spill kit, as mercury is not amenable to standard absorbent cleanup and requires specialized containment.*

### Animals

A total of four male mice aged 7–12 weeks (C57BL/6J-Tg­(Thy1-GCaMP6s)­GP4.3Dkim/J) obtained from the Jackson Laboratories were used for this study (stock# 024275). Mice were group-housed and the animal room was maintained at 22 ± 2 °C with 50% ± 10% humidity on a 12:12 light:dark cycle. Animals had ad libitum access to a standardized rodent diet and autoclaved water. Cages were provided with corncob bedding and environmental enrichment consisting of nesting material and a plastic shelter. All experiments were performed in accordance with the institutional guidelines set by the Institutional Animal Care and Use Committee of the University of Pittsburgh.

### Surgery and Electrode Implantation

Surgical preparations were as previously reported.^29^ Briefly, mice were anesthetized with 75 mg/kg ketamine and 10 mg/kg xylazine intraperitoneally throughout the surgical procedure and subsequent imaging. The depth of anesthesia was monitored through respiration rate and toe pinch response. A craniotomy was performed without durotomy. Craniotomies were centered at approximately 2.0 mm posterior and 2.0 mm lateral to bregma, targeting posterior primary somatosensory cortex. A single CFME was implanted into the cortex under 2P at 30° from horizontal using a microdrive (MO-81, Narishige, Japan) to a cortical depth of approximately 135 μm.

### Electrochemical Waveform Application

Within each electrode penetration, waveforms were applied in order of increasing intensity, beginning with standard literature waveforms and progressing to parametrically modified waveforms (increasing switching potential, altered scan rate, or frequency). Following evidence of stimulation-induced depression of neuronal excitability (SIDNE)^54^ (Figure 4A), the recording site was moved to a fresh cortical location with a new CFME. This approach was adopted to minimize confounds from repeated stimulation at a single site, leading to SIDNE.

FSCV scans were applied with an EI400 Potentiostat (out of production) using CV TarHeels software. SWV and amperometry were performed by using an Autolab PGSTAT128N potentiostat/galvanostat (Metrohm, Herisau, Switzerland) with Nova 2.1.4 software. To access raw SWV data, the analog current output of the Autolab potentiostat was connected to a PowerLab 4/26 data acquisition system (AD Instruments, Colorado Springs, CO). Analog data were digitized at 1000 Hz and exported to GraphPad Prism (GraphPad Software, Boston, MA) for analysis. Maximum potential excursions, scan rate, waveform application frequency, and duration of scan were all modified as indicated in the text to examine their effects on the tissue. The CFME served as the working electrode with an Ag/AgCl reference electrode and a platinum wire counter electrode placed into PBS on the surface of the skull. Because of noise caused by the 2P laser, the current data used to calculate thresholds were replicated in vitro for each waveform.

### 2P Imaging and Image Preprocessing

Fluorescence images were acquired with a 2P microscopy system (2P Plus, Bruker Nano, Inc.) equipped with a 16× 0.8NA objective lens (Nikon, Inc.) and a tunable laser (Insight X3, Newport Spectra-Physics, Inc.). All images were acquired at 512 × 512 pixel resolution, 1.3× zoom, and 2.4 μs dwell time, resulting in a field of view (FOV) of 867.46 μm × 867.46 μm and a frame period of 0.987 s. The FOV was roughly centered on the tip of the CFME electrode in *x*, *y*, and *z* planes with the *z* depth approximately 135 μm below the pia mater. GCaMP activity was imaged using 920 nm wavelength excitation. For each FSCV application, imaging began 30 s prior to stimulus onset and continued for at least 60 s after the application of FSCV was stopped (Figure 1A). For the no-FSCV control trials, the same imaging parameters were used and followed the same schedule as stimulus trials, but no waveform was applied. For all experiments, total laser power was kept below 30 mW. All images were imported into MATLAB, where motion correction and intensity correction were performed using custom scripts.

### Time Course of Activation

For each time series, in a 278 × 278 μm region of interest (ROI) centered on the electrode tip, the percent change of GCaMP activity in putative neurons was calculated as follows using a custom MATLAB script: The standard deviation projection of the time series was flattened using local contrast homogenization to account for inhomogeneities in local image intensity. A Laplacian of Gaussians filter was applied to detect putative GCaMP somata. The filtered image was thresholded to generate a neuron mask. The pixel intensity in each putative soma was counted as a neuron, and those pixels included in the neuron mask were used to calculate the average %dF/F using the mode as the baseline (Figure 3A–D).

### Area of Activation

For each time series, in a 278 × 278 μm region of interest (ROI) centered on the electrode tip, pixelwise *z*-scoring of GCaMP intensity was applied by calculating the mean intensity and standard deviation in the 30 s prestimulus baseline period, subtracting the mean and dividing the remainder by the standard deviation. Pixels with *z* > 3 for >5% of the imaging period were counted as activated and the total area was taken as the area of neural tissue activated by the application of the sensing waveform (Figure 1C).

Baseline sessions were collected where no stimulus was applied, and the area of activation using the z-score method was computed, and this was used as a noise control. For stimulus-present sessions, areas of activation greater than the mean +3 standard deviations of the area of activation in the noise control sessions were used as the threshold for a stimulus that strongly activated the tissue.

### Power and Duty Cycle Calculations

To estimate the electrical power delivered by each sensing waveform, we used simultaneously recorded voltage and current for a single scan. Instantaneous power was computed as *p*(*t*) = *V*(*t*)*i*(*t*), where *V*(*t*) is the applied waveform voltage and *i*(*t*) is the measured electrode current. Power per scan (*W*) was calculated as the sum of *p*(*t*) over the scan duration for each waveform condition. The duty cycle is defined here as the percentage of time the waveform was scanning vs at the holding potential. Power dosages were calculated by multiplying the power delivered by the scan with the duty cycle determined from its scan rate and application frequency.

### Electrochemical Equations

Faradaic current:

1 $$i_{peak\;faradaic}=2.69\times 10^{5}n^{3/2}AD_{O}^{1/2}C_{O}^{*}\nu^{1/2}$$

Non-Faradaic current:

2 $$\left|i_{non\text{‐}faradaic}\right|=AC_{d}\nu$$

### Statistical Analysis

The threshold for identifying a strong neural response was defined empirically using dedicated no-stimulation baseline imaging sessions collected at the same electrode penetration sites prior to the sensing waveform application sessions. For these control sessions, no sensing waveform was applied, and the T-Series collected were processed through the identical area-of-activation pipeline used for the sensing waveform application conditions (see Area of Activation above). The resulting distribution of activation areas under no-stimulation conditions had a mean of 287 μm^2^ and standard deviation of 229 μm^2^. We defined the threshold for a strong stimulus-evoked response as this empirical mean plus 3 standard deviations or an activation area of 974 μm^2^ or greater. This threshold is grounded in the Vysochanskij–Petunin inequality, which guarantees that, for any unimodal distribution with finite variance, the probability of a value falling 3 or more standard deviations from the mean is at most 4.9%,^55^ with a one-sided false-positive rate of ∼2.5% for approximately symmetric distributions. Group comparisons of activation area across conditions (standard dopamine and serotonin waveforms vs no-stimulation controls, Figure 2A) were performed using the Kruskal–Wallis test. The relationship between power dose and activation area (Figure 2E) was assessed using Pearson’s correlation coefficient. All statistical analyses were performed in Graphpad Prism.

**Figure 2:**
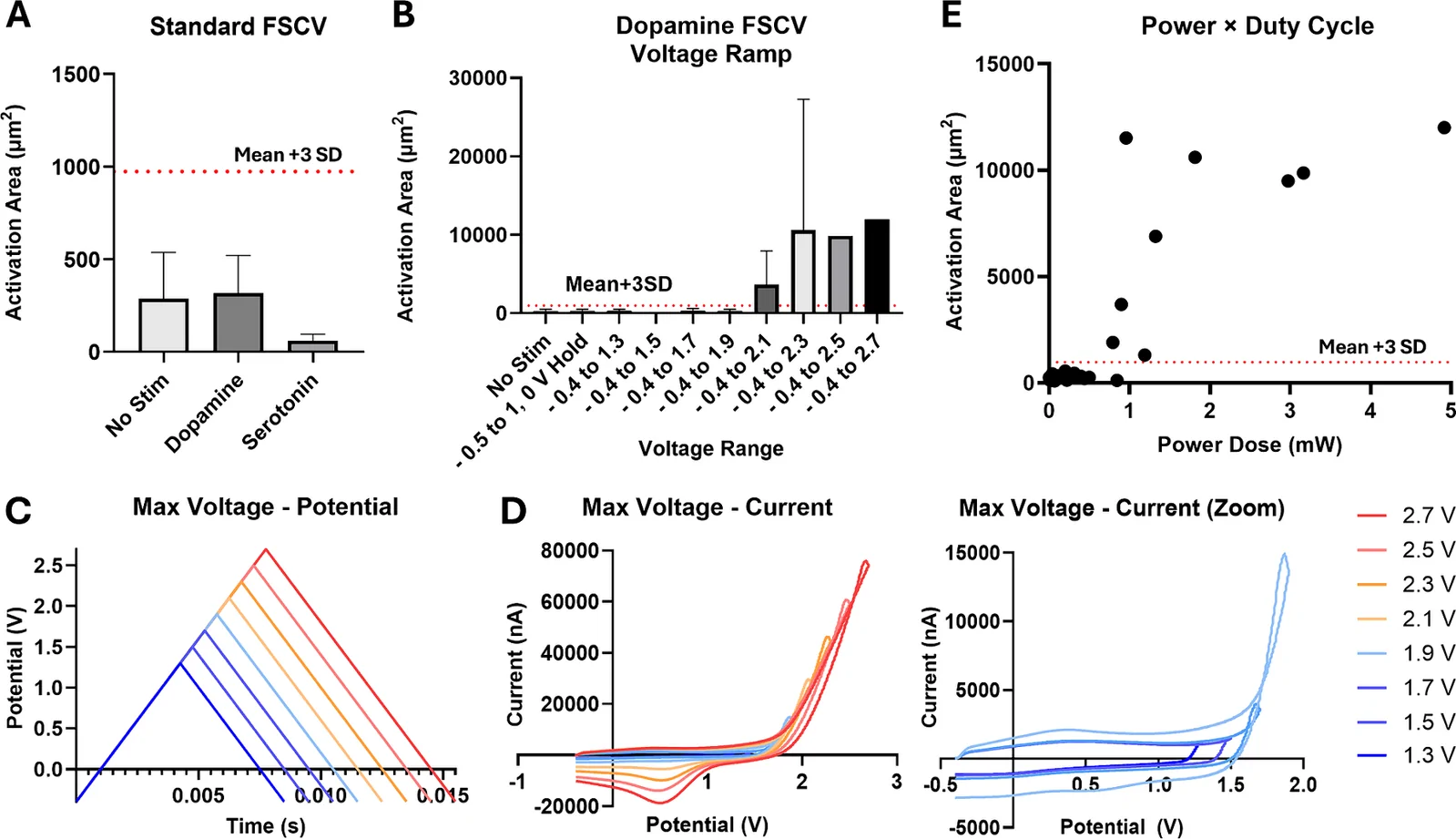
Neuronal response to standard neurochemical sensing waveforms and increased anodic switching potential. A) Area of Ca^2+^ activation in the tissue surrounding the CFME in no stim, 30 s standard dopamine, and 30 s standard serotonin FSCV application (Ns, *p* > 0.1, Kruskal–Wallis test, *n* = 6, 6, 4). B) Area of Ca^2+^ activation in the tissue surrounding the CFME in no stim, 30 s standard dopamine, and 30 s dopamine at increasing anodic switching potential in 0.2 V steps up to 2.7 V. C) Voltammogram of a single sweep of the dopamine detection waveform at a range of reversal potentials from 1.3 V (standard) to 2.7 V. D) Left: Cyclic voltammogram of the waveforms tested in B. Right: Cyclic voltammogram of the waveforms tested in B, scaled to provide detail for the lower voltages. E) Activation area for conditions tested in B by power dose (mW) calculated as power per scan × duty cycle (see Materials and Methods).

## Results

In these experiments, anesthetized mice with craniotomies were placed under a 2P microscope, and a single CFME was inserted into the cortex. Ca^2+^ imaging began 30 s prior to a 30 s application of the sensing waveform, followed by 1 min of postsensing-waveform imaging (Figure 1A; see Methods for details). An example waveform trace for the standard dopamine sensing waveform is shown in Figure 1B. To quantify the level of Ca^2+^ activation in the tissue, we first *z*-scored the pixel values and set a threshold of >3 standard deviations above baseline for >5% of frames for pixels to be included in the area of tissue activated by the sensing waveform application (Figure 1C; see Methods for details). An example of the large non-faradaic current generated by standard dopamine FSCV and the method of background subtraction that typically removes this current from the observed voltammogram is shown in Figure 1D.

### Standard Neurochemical Sensing Waveforms Do Not Strongly Activate Neural Tissue

We began this series of experiments by testing the application of widely used “standard” waveforms for detecting neurotransmitters via FSCV; details are in Table 1. The application of these standard waveforms led to a neural response that was indistinguishable from the no-stimulation baseline (Figure 2A). In addition to 30 s of continuous application, we tested longer applications including 5, 15, and 30 min since many neurochemical sensing experiments apply waveforms for longer durations, but even for these longer applications, we did not find any measurable increase in neural activation for the standard dopamine sensing waveform (Figure S1A). We also tested other standard FSCV waveforms for common analytes as well as SWV waveforms for dopamine and methylene blue-based aptamer sensors (Figure S1B) and the application of amperometry at the oxygen reduction (−0.6 V) and hydrogen peroxide oxidation (+0.7 V) potentials for 30 s and for 15 min (Figure S1C). For all these conditions, the neuronal Ca^2+^ response was indistinguishable from baseline.

**Table 1:** Standard FSCV Sensing Waveform Parameters

Holding Potential (V)	Anodic Switching Potential (V)	Cathodic Switching Potential (V)	Scan Rate (V/s)	Frequency (Hz)	Analyte(s)	Selected Citations
–0.4	1.3	N/A	400	10	Dopamine	^56^
0	1.0	–0.5	400	10	Dopamine	^57^
0.2	1.0	–0.1	1000	10	Serotonin	^58^
0.2	1.3	–0.1	1000	10	Serotonin	^59^
0.2	1.3	N/A	600	10	Melatonin	^60^

### Increasing Switching Potential Strongly Activates Neural Tissue

After the initial test of common standard waveforms already in use in the literature, we began systematically adjusting single waveform parameters independently while holding all others constant using the dopamine waveform, one of the most commonly used waveforms in the literature, as the base waveform. We did this to isolate thresholds for each parameter that would result in activation.^40^ We found that increasing the anodic switching potential of the dopamine sensing waveform by 0.2 V increments eventually led to strong neural activation >3 SD beyond the no stimulation baseline (Figure 2B). The voltage threshold to trigger this strong neuronal Ca^2+^ activation occurred at the 2.1 V anodic switching potential. Unlike the voltage steps, which increase monotonically (Figure 2C), the peak current generated by the waveforms measured in vitro increased nonlinearly (Figure 2D). The power dose (mW) also increased nonlinearly (Figure 2E) and closely tracked the neural response with a high correlation (*r* = 0.8419, *p* = 1.9582 × 10^–8^, *n* = 28, Pearson’s correlation). The neural response, however, appeared to saturate quickly, as there was little increase in the activation area beyond 2.3 V anodic switching potential.

### Elevated Neural Activity Following the Application of High Switching Potential Scans Is Delayed

For the conditions that elicited a strong neural response, we assessed the timing of the response by first taking the average %dF/F for neuron ROIs in the 278 μm ROI centered on the probe tip and across the full imaging time, including the 1 min (or longer) imaging period after the waveform application ended. A baseline (no stimulus control) recording is shown in Figure 3A. An example trace for a standard intracortical microstimulation (ICMS) pulse train delivered through an IrOx electrode at 40 μA current is shown for comparison, where the neuronal Ca^2+^ response occurred at the time stimulation was delivered and dissipated within seconds (Figure 3B). Application of the standard dopamine sensing waveform with the standard 1.3 V switching potential was indistinguishable from baseline (Figure 3C). In contrast to the ICMS pulse train, the response to the application of sensing waveforms with switching potentials high enough to generate a neural response was delayed from the onset of waveform application (Figure 3D). To assess the duration between the stimulus onset and the peak response, we computed the shift resulting in the maximum temporal cross correlation between the stimulus period and the average %dF/F trace (Figure 3E). For high anodic switching potentials (above 1.9 V), the average delay was 52 s (±26 s, *n* = 11) from waveform onset (Figure 3F).

**Figure 3:**
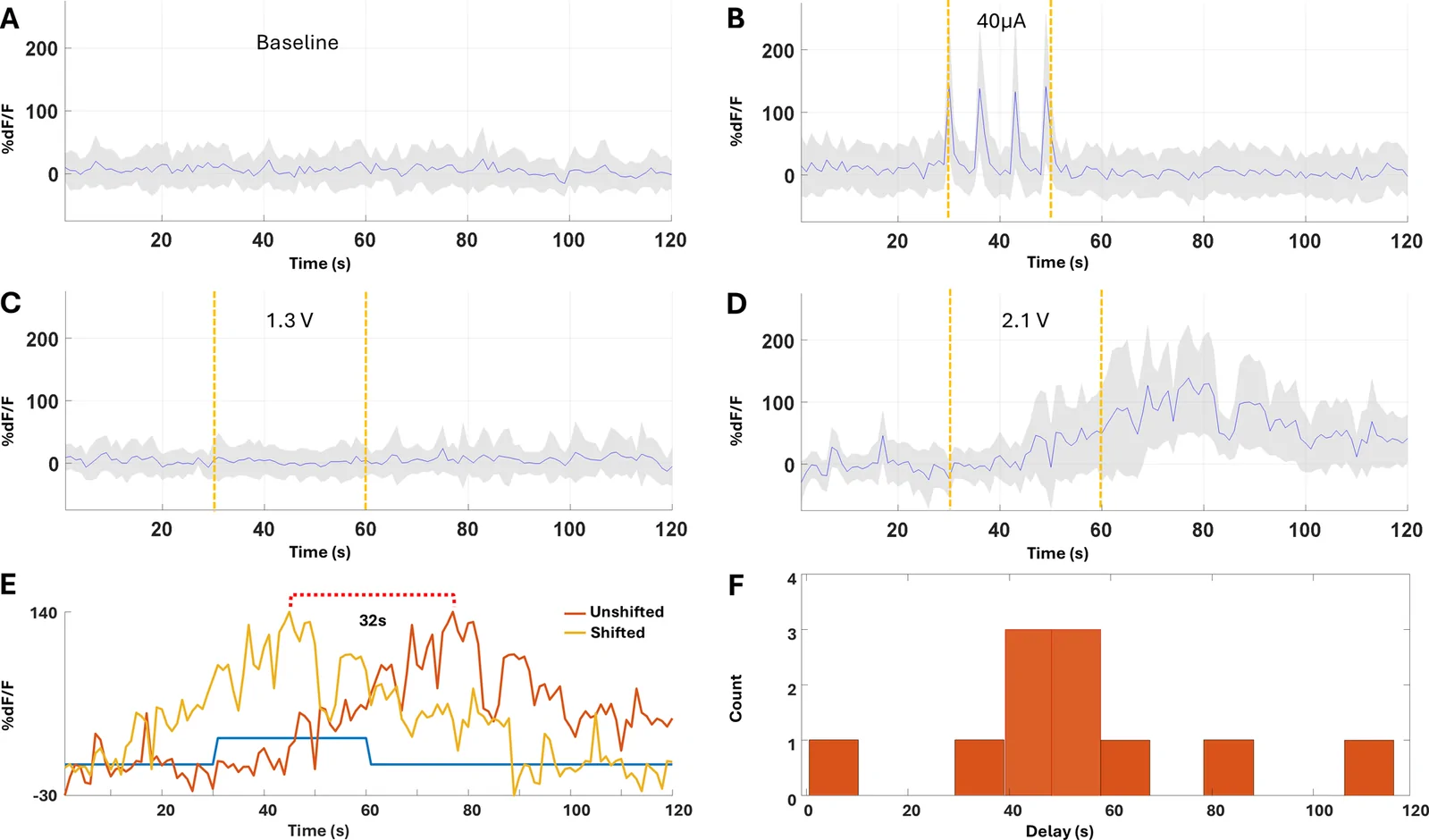
Time course of the neuronal response to standard neurochemical sensing waveforms at increased anodic switching potentials. A) Example %dF/F plot of the neural response during 120 s of baseline imaging (no stimulation control). B) Example %dF/F plot of the neural response to an intracortical microstimulation pulse train delivered at 40 μA, 4 pulse trains each 1 s at 50 Hz with 5 s between pulse trains. C) Example %dF/F plot of the neural response to a 30 s dopamine neurochemical sensing waveform application with the standard 1.3 V switching potential. D) Example %dF/F plot of the neural response to a 30 s dopamine neurochemical sensing waveform application with a 2.1 V switching potential. Gray ribbon represents 1 standard deviation above/below the mean (blue) in A–D. E) The %dF/F trace of the response from B (orange) with the shifted trace (yellow) that maximizes the temporal cross-correlation with the stimulus reference (blue). The offset between the original trace and the shifted trace represents the delay between stimulus onset and the maximal neural response. F) Histogram of the offsets computed using the method in C for all dopamine sensing waveforms at switching potentials that elicited a strong response (activation area >3 SD above the mean of the no stimulus control condition). The mean delay was 52 s ± 26 s (*n* = 11).

### High Switching Potentials Lead to Prolonged Periods of Elevated Neural Activity

Following the application of a dopamine-sensing waveform with an increased switching potential (2.5 and 2.7 V) eliciting a strong neural response, we performed an additional imaging scan immediately afterward in which no sensing waveform was applied. We found that, following the initial elevated neural response generated by the 2.5 V switching potential scan, the Ca^2+^ activity was near baseline levels in the 2 min following; however, following the 2.7 V switching potential scan, neuronal Ca^2+^ remained elevated for the 2 min after the sensing waveform application had ended (Figure 4A).

**Figure 4:**
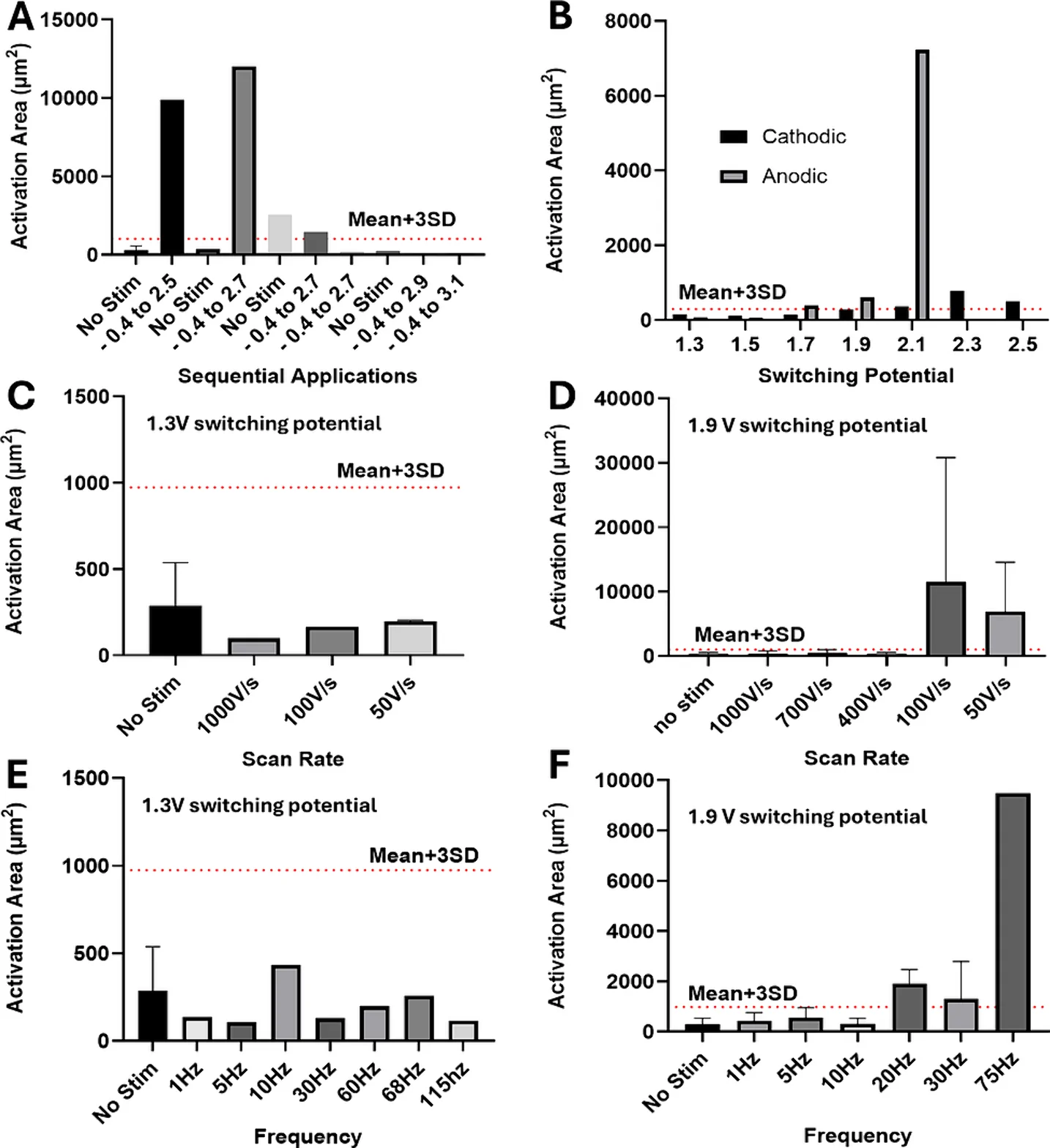
Effects of repeated stimulation, cathodic switching potentials, scan rate, and scan frequency on neural activation. A) Area of Ca^2+^ activation in the tissue surrounding the CFME in sequential applications of no stim, 30 s dopamine waveform at increased anodic switching potential at 2.5 V and increasing in 0.2 V steps up to 3.1 V, interleaved with 2 min no-stim (baseline) imaging sessions. The dashed line represents the mean + 3 standard deviations of the no stim condition. B) Area of Ca^2+^ activation in the tissue surrounding the CFME in sequential applications of 30 s dopamine FSCV at increased switching potentials from 1.3 V (standard) and increasing in 0.2 V steps up to 2.5 V for both anodic switching potentials (gray bars) and cathodic switching potentials (black bars). C) Area of Ca^2+^ activation for applications of 30 s of the standard dopamine FSCV across a range of different scan rates from 1000 V/s down to 50 V/s. D) Area of Ca^2+^ activation for applications of 30 s of the standard dopamine FSCV at 1.9 V switching potential across a range of different scan rates from 1000 V/s down to 50 V/s. E) Area of Ca^2+^ activation for applications of 30 s of the standard dopamine FSCV across a range of different scan frequencies from 1 Hz up to 115 Hz. F) Area of Ca^2+^ activation for applications of 30 s of the standard dopamine FSCV at 1.9 V switching potential across a range of different scan frequencies from 1 Hz up to 75 Hz.

### Repeated Applications of High Switching Potential Scans Lead to Stimulation-Induced Depression of Neuronal Excitability

After these prolonged periods of elevated neural activity, subsequent sensing waveform application at switching potentials that previously elicited a strong response (2.7 V), and at even higher switching potentials (up to 3.1 V), failed to elicit a response (Figure 4A). Following these instances of SIDNE, the site of stimulation was moved to a fresh cortical location with a new CFME for subsequent sensing waveform applications.

### Anodic Switching Potentials Lead to Stronger Neural Activation

In addition to experiments testing the effect of increasing the anodic switching potential, we also tested sensing waveforms in which the maximum voltage excursion was cathodic (e.g., methylene blue reported aptamer sensing SWV) as well as some FSCV waveforms mimicking the dopamine FSCV parameters but with a cathodic rather than anodic switching potential. In all cases, strong neural activation was observed at lower voltages for anodic than for cathodic switching potentials (Figure 4B).

### Slower Scan Rates Lead to Stronger Neural Activation

In addition to switching potential, we also parametrically tested the effect of altering the scan rate on the neural response. At the standard switching potential for dopamine sensing FSCV (1.3 V), there was no effect of altering scan rate between 50 V/s and 1000 V/s as none of the scans elicited a neural response above baseline (Figure 4C). However, at a somewhat increased switching potential (1.9 V) that was still below the threshold for strong neural response at the standard scan rate of 400 V/s, an effect of scan rate was observed whereby decreasing scan rate to 100 V/s and below did elicit a strong neural response, whereas increasing scan rate above the standard 400 V/s (up to 1000 V/s) did not (Figure 4D).

### Higher Scan Frequencies Lead to Stronger Neural Activation

In addition to testing the effects of parametric scan rate modulation, we also tested the effect of parametrically modulating the scan frequency on neural activation. As with the scan rate experiments, at the standard switching potential for dopamine sensing FSCV (1.3 V), there was no effect of altering scan frequency between 1 and 115 Hz, as none of the scans elicited a neural response above baseline (Figure 4E). At the moderately increased switching potential (1.9 V), however, an effect of scan frequency was observed whereby increasing scan frequency to 20 Hz (from the standard 10 Hz) and above (up to 75 Hz) did elicit a strong neural response, whereas decreasing the scan frequency below the standard 10 Hz did not (Figure 4F).

### Trends in the Neural Activation Profile Generalize to SWV-Based Sensing

Similar to FSCV, in SWV-based sensing, the displayed signal typically appears far smaller and far smoother than the raw current to the user, depending on the software used (Figure 5A). We began by testing a SWV sensing waveform for methylene blue-based aptamer sensors^61–63^ and a waveform for the detection of dopamine.^64,65^ The parameters are shown in Table 2. As with the standard FSCV waveforms, no detectable Ca^2+^ activity was observed for these waveform applications (Figure S1B). As with FSCV, a similar activation threshold and nonlinear response were seen when we increased the anodic potential maximum for the dopamine sensing SWV waveform (Figure 5B) with the threshold for strong activation between 1.5 and 2.5 V for the dopamine SWV waveform. We also observed the prolonged activation in no-stim trials following strong activation, as seen with FSCV. We also tested increasing the maximum voltage for the methylene blue-based aptamer SWV waveform, which uses a cathodic potential step, and found that the threshold for strong activation was between −2.3 and −2.5 V (Figure 5C).

**Figure 5:**
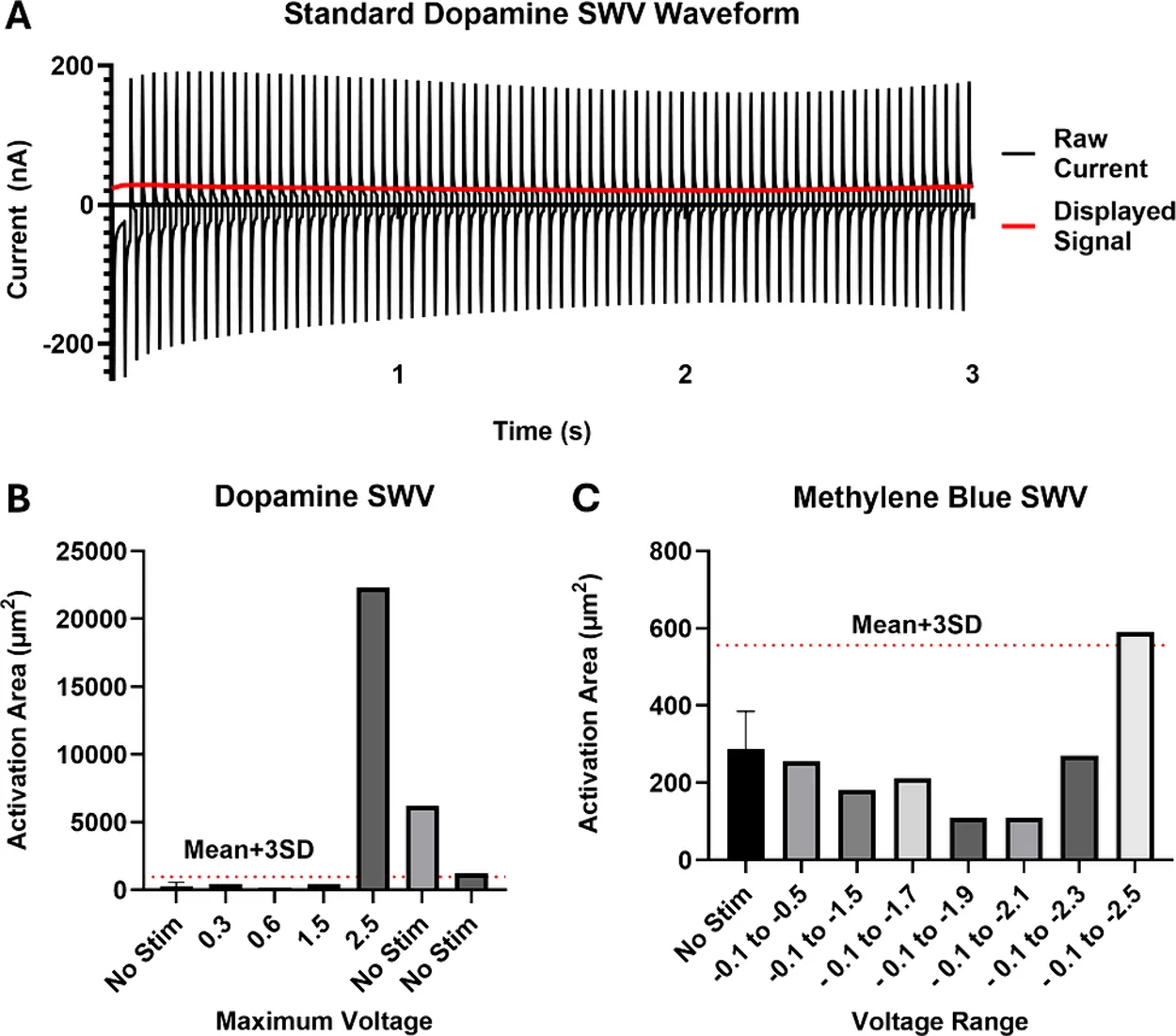
Neuronal response to SWV sensing waveforms at increased anodic and cathodic potentials. A) Example dopamine square wave voltammogram, showing the large non-faradaic current (black trace) compared to the displayed dopamine signal (red trace). B) Area of activation for the square wave voltammetry waveform at the standard +0.3 V maximum potential and incrementally increasing maximum potentials up to +2.5 V. C) Methylene blue aptamer-based SWV applied at the standard −0.5 V maximum potential and incrementally increasing maximum cathodic potentials up to −2.5 V.

**Table 2:** Standard SWV Sensing Waveform Parameters

Start Potential (V)	Stop Potential (V)	Step (V)	Modulation Amplitude (V)	Frequency (Hz)	Analyte	Selected Citations
–0.1	–0.5	–0.005	–0.025	100	Methylene blue	^66^
–0.2	0.3	0.005	0.05	25	Dopamine	^64^

## Discussion

In this study, we investigated whether neurochemical sensing waveforms alter local neuronal activity in vivo. FSCV is typically treated as a measurement-only technique, even though it applies large, rapidly changing voltages repeatedly and produces substantial non-faradaic current that must be subtracted and, depending on the software, may not even be easily visible to the experimenter.^40^ For similar reasons, we investigated SWV as it is a technique that also involves rapidly changing potentials that induce large amounts of current that are often “hidden” from the user by automated software calculations. Finally, we included amperometry for its relevance in clinical research and patient care.^67–69,9^ Here, by combining in vivo 2P Ca^2+^ imaging in Thy1-GCaMP6s mice with controlled-voltage waveform application through a CFME, we show two main findings. First, standard, widely used sensing waveforms, including standard dopamine and serotonin FSCV settings and several SWV and amperometric methods, do not strongly activate local cortical neurons above baseline. Second, shifts away from standard parameter regimes, especially those that increase potential excursions and increase the amount of time spent at extreme potentials, can produce strong neural activation but with delayed kinetics that appear distinct from conventional intracortical microstimulation.

### Standard Sensing Waveforms Do Not Strongly Activate Cortical Tissue under These Conditions

Across the standard FSCV waveforms tested here, we did not observe an increase in the Ca^2+^ activation area beyond the no-stimulation baseline. This was true for the typical 30 s application and also for substantially longer applications of the standard dopamine waveform. This provides practical evidence that the parameter regimes most commonly used for in vivo FSCV sensing, especially the standard dopamine waveform highlighted in foundational and modern FSCV work,^23,40^ do not produce strong neuronal Ca^2+^ activation in the superficial cortex under anesthesia. Importantly, our results support the common assumption that FSCV measurements under established parameters are not typically confounded by waveform-evoked neural activity that would be folded into the background signal and removed by subtraction. Our findings indicate that, within the standard parameter space, these sensing waveforms can be considered physiologically quiet in the sense that they do not generate a strong neuronal Ca^2+^ response detectable at the scale measured here. Although direct empirical studies of the effect of neurochemical sensing waveforms on neuron dynamics remain rare, our results are consistent with a previous study demonstrating the lack of changes to spiking activity induced by FSCV using patch clamp recordings in rat striatum slices.^49^

### Large Potential Excursions Drive Activation in a Dose-Dependent Manner

When we increased the anodic switching potential of the dopamine sensing waveform, we observed a clear transition from baseline-like activity to strong activation with an apparent threshold around 2.1 V under our conditions. The response also appeared to saturate quickly with minimal further increases in activation beyond 2.3 V.

Increasing the switching potential is commonly considered to increase sensitivity and modify the carbon surface. Our results show that, in vivo, increasing switching potential can move a waveform into a regime that strongly activates neural tissue. This point is particularly relevant, as nonstandard waveforms are increasingly used to detect additional targets.

We also compared anodic and cathodic switching potential variants and found that strong activation occurred at less extreme voltages for anodic switching potentials than for cathodic switching potentials. This asymmetry suggests that the underlying driver of activation is not simply the magnitude of the voltage excursion but depends on the polarity-specific processes engaged by extreme anodic and cathodic excursions in brain tissue.

At a standard switching potential (1.3 V), varying scan rate and scan frequency across a wide range did not produce strong activation, consistent with our initial conclusion that standard waveforms are generally quiet. However, at a moderately elevated switching potential (1.9 V), we observed that slower scan rates (≤100 V/s) and higher scan frequencies (≥20 Hz) were more likely to produce strong activation. These trends are consistent with the interpretation that neural activation depends not only on the maximum voltage but also on the exposure per unit time, including the amount of time spent away from the holding potential during each scan (influenced by scan rate and waveform shape) and the number of scans delivered per unit time (scan frequency). Slower scans increase the time spent at more extreme voltages per scan, and higher scan frequency increases the number of scans delivered per second. Together, these effects work to increase the total effective dose of the applied waveform.

### Repeated High Switching Potential Applications Can Produce a Depression of Subsequent Responsiveness

Following strong activation induced by high switching potentials, subsequent applications of waveforms that previously elicited strong responses sometimes failed to elicit a response. This is consistent with SIDNE as reported in the classic ICMS literature,^54^ although the mechanisms driving the effect are likely different for the neurochemical sensing waveforms used here. This has implications for studies that apply sensing waveforms repeatedly over long periods, particularly if waveforms are being pushed toward extreme switching potentials.

### The Delayed, Slow Time Course Is in Contrast to Instantaneous Direct Electrical Stimulation-Induced Activation

A defining feature of the neural responses we observed here was the pronounced delay between waveform onset and the peak Ca^2+^ response. For waveforms employing elevated switching potentials, the peak response often occurred tens of seconds after onset with an average delay of approximately 52 s. This temporal profile differed qualitatively from the Ca^2+^ response evoked by a control ICMS pulse train, which occurred during stimulus delivery and decayed within seconds.

This delayed time course, together with the persistence of activity after waveform cessation, argues against direct millisecond-scale membrane depolarization as the primary mechanism. In conventional ICMS, cathodic-leading pulses generate a local current sink that depolarizes nearby membranes and produces responses tightly time-locked to the stimulus train. In contrast, we found that anodic excursions were more effective than cathodic excursions in eliciting robust Ca^2+^ signals and that these signals emerged only after tens of seconds. These observations instead support a mechanism that accumulates over repeated scans, progressively altering local tissue excitability, until a threshold for broader network activation is reached.

At sufficiently positive potentials in a chloride-containing electrolyte, anodic oxidation of chloride can produce chlorine, which hydrolyzes to hypochlorous acid and related reactive chlorine species. While the precise potential at which these reactions begin to occur depends on the specifics of the electrode material and electrolyte composition, published measurements on carbon-based anodes in PBS are on the order of 1.1 to 1.4 V vs Ag/AgCl,^70^ well below the +2.1 to 2.7 V that produced strong activation in our experiments. Additionally, acidosis generated by water oxidation at the electrode can increase intracellular Ca^2+^ via acid-sensing ion channel 1a (ASIC1a), which mediates an acidosis-induced Ca^2+^ influx and elevation of neuronal [Ca^2+^].^71^

Overall, our observations are most consistent with electrochemically driven chemical changes at the electrode–tissue interface, including pH/ionic shifts and the possible formation of reactive species (notably reactive chlorine), followed by diffusion-limited spread and network recruitment,^54,70,71^ leading to our observed profile of little immediate response at waveform onset, followed by a slow ramp and slow recovery after waveform cessation.

### Implications for Waveform Development and in Vivo Neurochemical Measurements

These findings have important implications for ongoing efforts to design and implement new waveforms for in vivo neurochemical sensing.^13,40^ FSCV is inherently a differential technique and relies on the subtraction of large background currents. If a sensing waveform were to induce neurotransmitter release or broader network activation, then it would introduce confounding changes in the measured electrochemical signal that could be difficult to distinguish from true analyte dynamics. Needless to say, such neural activation would perturb the underlying neurophysiological and neurochemical milieu that one intended to study.

Our results indicate that widely accepted waveforms for common analytes are unlikely, under our experimental conditions in the cortex, to produce such confounds. However, waveform modifications intended to enhance sensitivity, such as increasing switching potential or scan frequency and decreasing scan rate, can shift operation into a regime in which neural activation becomes more probable. The waveforms that produced tissue activation in the present study involved parameter extremes that are not typical of those of standard in vivo FSCV experiments. Nevertheless, prior reports have described in vitro and in vivo waveforms with unusually long duty cycles or large voltage excursions.^42,46^

Accordingly, the experiments presented here are intended to delineate a threshold regime for neural activation, providing practical guidance for investigators developing new waveform paradigms and highlighting the importance of considering potential bioelectrical and electrochemical side effects during waveform optimization.

To inform waveform development, we sought to define a quantitative threshold above which a waveform is more likely to activate the neural tissue. Specifically, we evaluated the combined effect of scan power and duty cycle by defining a *power dose* as the power delivered per scan multiplied by the duty cycle. For waveforms that included an anodic excursion, we found that a power dose exceeding 0.5 mW was associated with neural activation in nearly every case. These findings suggest that waveform optimization should account not only for analytical performance (e.g., sensitivity and selectivity) but also for physiological impact in vivo. In particular, estimating the predicted power dose from in vitro measurements may provide a practical framework for assessing the likelihood of tissue activation prior to its in vivo implementation.

### Limitations and Future Directions

This study has several limitations that define clear directions for future work. First, all experiments were performed using CFMEs as the working electrode. CFMEs are the most widely used electrode material for in vivo neurochemical sensing, and the standard waveforms benchmarked here (Tables 1 and 2) were themselves developed and validated using this electrode type. In addition, the ultrasmall footprint and ease of insertion of CFMEs make them particularly well suited for the two-photon imaging experiments that form the primary experimental platform of this study. However, electrode material influences the overpotential for water oxidation and chloride hydrolysis. Other electrode materials commonly used for neurochemical sensing, including glassy carbon, carbon nanotube-modified surfaces, and noble metals such as platinum, have distinct water oxidation and chloride hydrolysis potentials and may therefore exhibit different tissue activation thresholds and power-dose relationships than those reported here. The power-dose threshold we identify (∼0.5 mW) should therefore be understood as specific to CFMEs. A systematic comparison across electrode materials represents an important direction for future work.

Second, while we systematically varied several individual waveform parameters, including anodic and cathodic switching potential, scan rate, and scan frequency, other waveform features such as holding potential were not independently varied across their full range in this study. Future work testing established FSCV waveforms for additional analytes such as hydrogen peroxide and adenosine and systematically isolating additional waveform parameters could further refine guidelines for designing sensing waveforms that avoid neural activation. The data set and power-dose framework established here may also serve as a foundation for computational approaches, such as Bayesian optimization-guided waveform design as recently demonstrated for serotonin-selective voltammetry waveforms,^72^ machine learning, principal component analysis, or regression-based modeling to predict activation risk directly from waveform parameters and guide the design of novel, physiologically silent sensing waveforms.

Third, it is important to note that all our activation measurements were obtained in the cortex. Because thresholds of excitability vary across brain regions, it remains to be determined whether the threshold identified here is generalizable to other brain regions including subcortical structures such as the striatum or ventral tegmental area–nucleus accumbens, which are frequently targeted in neurochemical sensing studies. Imaging such deep structures would require either three-photon imaging or a chronic preparation incorporating a relay lens system (e.g., GRIN lens or microprism^73^).

Fourth, we chose to focus our experiments and analysis on oxidizing waveforms because of their preponderance in the literature. Cathodic waveforms with a cathodic sweep activated the tissue at a higher threshold than those with an anodic sweep. We therefore caution that there is likely a different power dose threshold for tissue activation for reducing waveforms that warrants a future systematic characterization.

Finally, our study focused exclusively on neuronal Ca^2+^ activation as the outcome measure and did not assess the impact of sensing waveforms on non-neuronal cells or the local vasculature. Glial cells, including astrocytes and microglia, are highly sensitive to shifts in extracellular pH and reactive oxygen or chlorine species and could plausibly be perturbed at switching potentials at or below the thresholds reported here. Electrochemically generated reactive species and local pH shifts may also affect the vascular tone and blood flow. Given the established sensitivity of both glial and vascular responses to electrical stimuli,^74^ studies systematically characterizing the impact of sensing waveforms on non-neuronal cells and vasculature represent an important direction for future work.

## Supplementary Material



## Notes

Supplemental Figure S1: Area of Ca^2+^ activation for standard dopamine FSCV applied across durations (5–30 min), standard literature waveforms, and amperometry at oxygen and hydrogen peroxide detection potentials (PDF)
