# Supplementary material for: Two-Photon Imaging Reveals a Power Threshold of Neuronal Activation by Electrochemical Sensing Waveforms
Source: Anal Chem. 2026 Aug 31;98(36):26649–60. doi: 10.1021/acs.analchem.6c03024 (PMC13584698; doi:10.1021/acs.analchem.6c03024)
Supplement: Supplementary file 1 [file ac6c03024_si_001.pdf]

Supplemental Information

Two-photon imaging reveals a power threshold of neuronal activation by electrochemical sensing waveforms

Nathaniel P. Williams[1,2], Elaine M. Robbins[1], Anna M. Kelly[1], Alberto L. Vazquez[2,3,4], X. Tracy Cui[1,2,4,*]

[1] Department of Bioengineering, University of Pittsburgh, Pittsburgh, PA 15260, United States.
[2] Center for Neural Basis of Cognition, University of Pittsburgh and Carnegie Mellon University, Pittsburgh, PA 15260,United States.
[3] Department of Radiology, University of Pittsburgh, Pittsburgh, PA 15260, United States.
[4] McGowan Institute for Regenerative Medicine, University of Pittsburgh, Pittsburgh, PA 15260, United States.
*Corresponding Author: X. Tracy Cui xic11@pitt.edu

Table of Contents

S1 Cover Page

S2 Figure S1

Figure S1

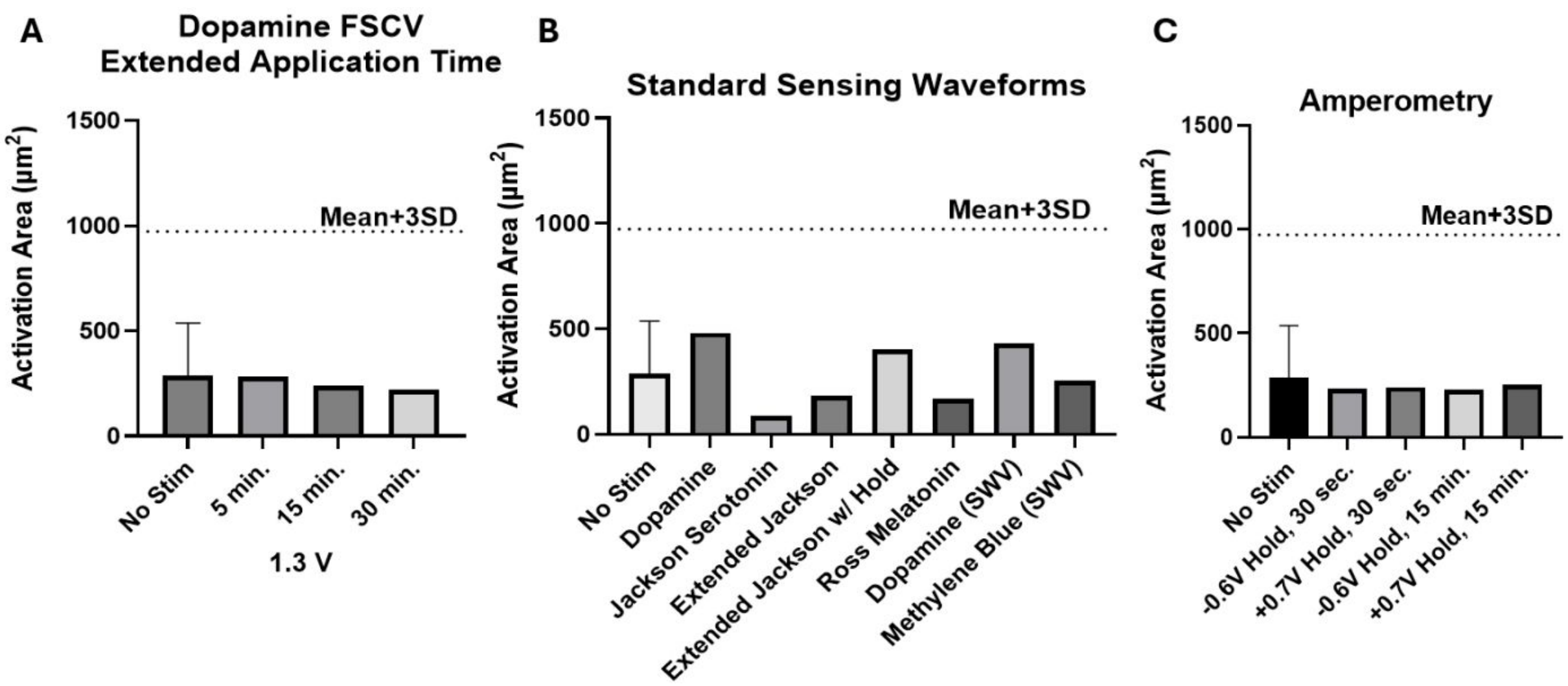

Supplemental Figure 1. A) Area of Ca^{2+} activation by thetandard dopamine sensing FSCV applied for varying durations from 5 to 30 minutes. **B)** Area of Ca^{2+} activation for each of the "standard" waveforms from the literature. **C)** Area of Ca^{2+} activation in the tissue surrounding the CFME in no stim and 30 s. and 15 min. applications of amperometry at the oxygen (-0.6V) and hydrogen peroxide (+0.7V) detection potentials.
